# Molecular and Functional Characterization of *CaNAC035*, an NAC Transcription Factor From Pepper (*Capsicum annuum* L.)

**DOI:** 10.3389/fpls.2020.00014

**Published:** 2020-02-04

**Authors:** Huafeng Zhang, Fang Ma, Xinke Wang, Suya Liu, Ul Haq Saeed, Xiaoming Hou, Yumeng Zhang, Dan Luo, Yuancheng Meng, Wei Zhang, Khan Abid, Rugang Chen

**Affiliations:** State Key Laboratory of Crop Stress Biology for Arid Areas, College of Horticulture, Northwest A&F University, Yangling, China

**Keywords:** pepper, NAC transcription factor, *CaNAC035*, abiotic stress, protein–protein interaction

## Abstract

NAC (NAM, ATAF1/2, and CUC2) proteins are the plant-specific transcription factors (TFs) which are important in plant response to abiotic stresses. However, knowledge about the functional role that NACs play in pepper abiotic stress tolerance is limited. In this study, we isolated a NAC TF gene, *CaNAC035*, from pepper (*Capsicum annuum* L.), where the protein is localized in the nucleus and functions as a transcriptional activator. *CaNAC035* expression is induced by low and high temperatures, osmotic stress, salt, gibberellic acid (GA), methyl-jasmonic acid (MeJA), salicylic acid (SA), and abscisic acid (ABA). To understand the function of *CaNAC035* in the abiotic stress responsep, we used virus-induced gene silencing in pepper to knockdown the *CaNAC035* and overexpressed the *CaNAC035* in *Arabidopsis*. The results showed that pepper seedlings in which *CaNAC035* was silenced, showed more damage than the control pepper plants after cold, NaCl, and mannitol treatments. Correspondingly increased electrolyte leakage, a higher level of malondialdehyde (MDA), H_2_O_2_, and superoxide radicals were found after cold treatments. *CaNAC035*-silenced seedlings exhibited lower chlorophyll content while *CaNAC035*-overexpressed *Arabidopsi*s plants had higher germination rate and fresh weight after mannitol and NaCl treatments. We also reported 18 proteins that potentially interact with CaNAC035 and may participate in processes such as the stress response, resistance, and photosynthesis. Our results suggest that *CaNAC035* is a positive regulator of abiotic stress tolerance in pepper which acts through multiple signaling pathways.

## Introduction

As sessile organisms, plants are continuously exposed to adverse external stimuli during their life cycle. Biotic (bacteria, fungi, viruses, and nematodes) and abiotic stresses (high/low temperatures, drought, and salinity), alone or in combination cause unfavorable conditions for plant growth and development, which results in changes in physiological, morphological, and/or biochemical reactions. (Meng et al., 2013). Some macromolecules including lipids and proteins are hydrolyzed and destroyed, which leads to metabolic disorders and death (Apse and Blumwald, 2002). In addition, the accumulation of more reactive oxygen species (ROS), can further accelerate the plant death (Bartels and Sunkar, 2005).

Transcription factors (TFs) are trans-acting proteins that act as molecular switches to regulate the expression of a variety of target genes by interacting with the *cis*-elements in the gene promoters (Singh et al., 2002). More than 50 TF families have been identified, based on bioinformatic analyses. Most plant TFs belong to large gene families, such as *WRKY*, *bZIP*, *NAC*, *AP2/EREBP*, and *bHLH*. Some TF family members are involved in the adaptive stress response, activating or inhibiting the expression of stress-related genes to alter the plant’s ability to adapt to stressful environments, while others help to coordinate the plants growth and development (Zhong et al., 2012). Members of the plant-specific NAC TF family [named for cup-shaped cotyledon (CUC2), *Arabidopsis thaliana* transcription activation factors (ATAF1/2), and no apical meristem (NAM)] are well known for their functional roles.

The NAC TF family is widely distributed in many land plant species (Shen et al., 2009). Since the first report of NAC TFs in *Arabidopsis* (Aida et al., 1997), research has progressed rapidly, mainly due to the availability of several complete plant genome sequences. Several members of the NAC family have been identified and characterized in plants such as *A. thaliana*, *Populus trichocarpa* (poplar), *Glycine max* (soybean), *Hordeum vulgare* (barley), *Triticum aestivum* (wheat), *Oryza sativa* (rice), and pepper (*Capsicum annuum*) (Ooka et al., 2003; Fang et al., 2008; Hu et al., 2010; Xia et al., 2010; Christiansen et al., 2011; Le et al., 2011; Diao et al., 2018). The typical structural characteristics of NAC proteins have been described and a few variations in the recognition of DNA binding (DB) sites in the target genes have also been identified (Olsen et al., 2005; Jensen et al., 2010). The typical NAC protein is characterized by a highly conserved NAC domain at its N-terminal and a highly divergent transcription regulatory region (TRR) at its C-terminal (Mao et al., 2007). The NAC domain comprises of about 160 amino acid residues that confer DB ability and/or are responsible for the protein binding and dimerization and can be divided into five conserved subdomains (A–E) (Hao et al., 2010; Cenci et al., 2014). The TRR domain functions as an activator or repressor and sometimes exhibits protein-binding activity (Kim et al., 2007). At least 13 NAC family members in *Arabidopsis* contain strong α-helical trans-membrane motifs at their C-termini, which are referred to as NTL. Most putative membrane-associated NAC TFs are closely associated with plant responses to various abiotic stresses. A phylogenetic analysis of 75 NAC proteins from rice and 105 NAC proteins from *Arabidopsis* classified the NAC domains into two groups: group I comprise 14 subfamilies including TERN, NAC1, SENU5, ANAC011 AtNAC3, ATAF, NAC2, NAP, TIP, OsNAC3, OsNAC8, OsNAC7, ONAC022, and NAM. The group II comprises four clades; among which ANAC001 and ANAC063 consist entirely of NAC TFs from *Arabidopsis* whereas the members of subgroups ONAC001 and OsNAC3 are from rice. Recently, a new subfamily termed as TNAC was identified in tobacco and it appears to be specific to the *Solanaceae*. The TNAC proteins lack the LPPG and YPNG motifs at the N-terminus of the NAC domain that is conserved in most NAC family members, and the conserved D/EEE motifs found in other NACs is replaced by D/ExE (Rushton et al., 2008; Singh et al., 2013).

Although numerous NAC genes have been identified in various plant species, only a few members of NAC proteins have been described for their biological functions, more than 90% still to be characterized (Lu et al., 2007). NAC TFs function in a wide range of biological and physiological processes including the formation and maintenance of shoot apical meristem (Souer et al., 1996; Nikovics et al., 2006), regulation of secondary cell wall synthesis (Mitsuda et al., 2007), hormonal regulation, signal transduction (He et al., 2005), mineral nutrition, nutrient substance transport (Uauy et al., 2006), floral and embryo development (Sablowski and Meyerowitz, 1998; Xie et al., 2000), lateral root development, and leaf senescence (Kim H.J. et al., 2014; Kim S. et al., 2014; Garapati et al., 2015). Numerous NAC TFs play crucial roles in regulating plant tolerance to biotic and abiotic stresses using transgene technology or microarray analysis. For instance, the function of ATAF1 in the drought tolerance was revealed based on the studies with *ataf1-1* and *ataf1-2* mutants and were confirmed by analysis of plants overexpressing *ATAF1.* Interestingly, overexpression of *ATAF1* also resulted in increased sensitivity to oxidative stress, the necrotrophic fungus *B*, abscisic acid (ABA), and high-salt conditions (Wu et al., 2009). In addition, expression of *ANAC019*, *ANAC055*, and *ANAC072* were induced by low temperatures, salinity, and drought stresses, and the *Arabidopsis* plants overexpressing these genes exhibited improved drought tolerance as compared to the wild type (WT) plants (Bu et al., 2008). *ANAC072* (RD26) is involved in a novel ABA-dependent signaling pathway (Fujita et al., 2004). *OsNAC6/SNAC2* transgenic lines exhibited tolerance to dehydration, salt stress, and blast disease, as well as improved the grain yield and *OsNAC6* transcription, was induced by abiotic stresses and jasmonic acid treatments (Rachmat et al., 2014). Furthermore, transgenic *Arabidopsis* overexpressing *SlNAC1* from *Suaeda liaotungensis* Kitag showed enhanced tolerance to drought, salt, and cold stresses (Li D.K. et al., 2014; Li X.L. et al., 2014). Recently, expression of *TaNAC69* in transgenic wheat plants driven by either the constitutive *HvDhn8s* promoter or the drought-inducible *HvDhn4s* promoter from barley significantly improved tolerance to mild salinity and polyethylene glycol-induced dehydration (Xue et al., 2011). Since, previous studies on NACs have mainly been performed in model plants such as *Arabidopsis* and rice, it is important to investigate the functions of NACs in other crops to uncover the common and specific roles of these crucial TFs in plant development and the stress response.

Pepper (*C. annuum* L.) is an important crop used as a seasoning vegetable that is widely cultivated in subtropical and temperate regions worldwide. Various abiotic stresses such as temperature extremes, drought, and high salinity, are major factors that influence the distribution, growth, and development of pepper plants. Therefore, it is important to investigate the mechanisms underlying resistance or tolerance in pepper to the adverse environmental conditions (Guo et al., 2015). In this study, a NAC transcription factor gene (*CaNAC035*) was isolated from pepper line ‘P70’ leaves. To elucidate the possible role of *CaNAC035* in response to salt, cold, and osmotic stresses, the *CaNAC035* was successfully knockdown in pepper plants through virus-induced gene silencing (VIGS) and overexpressed in *Arabidopsis* plants. The results divulged that *CaNAC035*-silenced pepper plants exhibited less tolerance to salt, osmotic and cold stresses, whereas *CaNAC035*-overexpressed *Arabidopsis* plants displayed a significant increase in the tolerance to the abovementioned abiotic stresses. The yeast two-hybrid (Y2H) screen identified 18 proteins, which potentially interacted with *CaNAC035*, and these 18 proteins are predicted to participate in several different biological processes. Taken together, these results suggest that *CaNAC035* positively regulates the abiotic stress tolerance and is involved in many different signaling pathways. This study provides valuable information regarding the function of this important gene family in pepper and other important crops.

## Materials and Methods

### Plant Materials and Treatments

Pepper plants ‘P70’ were provided by the pepper group, Northwest A&F University, China. The growth conditions were the same as described by Chen et al. (2014). Abiotic stresses (salt, heat, osmotic, and cold) and phytohormone treatments [ABA, methyl-jasmonic acid (MeJA), and salicylic acid (SA)] were applied as reported previously (Chen et al., 2015; Chen et al., 2019). For gibberellic acid (GA) treatment, GA (100 μM) with 0.5% Tween-20 were applied through foliar spray, while control plants were sprayed with sterile distilled water. The leaves were collected at 0, 1, 3, 6, 12, 24, and 48 h post-treatment (hpt), immediately frozen in the liquid nitrogen and stored at -80°C. Each treatment was performed with three independent biological replicates, and the samples were collected from three to five seedlings for each treatment at each replication.

### Sequences and Phylogenetic Analyses of *Canac035*

The nucleic acid sequence of *CaNAC035* was downloaded from the pepper genome PGD1 and PGP2 (Kim H.J. et al., 2014; Kim S. et al., 2014; Qin et al., 2014). According to the nucleotide sequences, the specific primers of *CaNAC035*-F1 (5ʹ-GCTCTAGAGGAGATTTATTCAGACGCCTT-3ʹ *Xba*I site underline) and *CaNAC035*-R1 (5ʹ-GGGGTACCGACCTACACCACCAAAGAAC-3ʹ *Kpn*I site underline) were designed to obtained the ORF sequence of *CaNAC035* from ‘P70’ pepper leaves, the PCR product obtained was subsequently sequenced. DNA from the leaves of pepper ‘P70’ seedlings was extracted through CTAB method.

The protein sequences from other different crop species were downloaded from GenBank (https://www.ncbi.nlm.nih.gov/genbank/). The alignment of full-length NAC amino acid sequences were performed by DNAMAN (Version 5.2.2.0, Lynnon Biosoft, USA). To examine the phylogenetic relationship, an un-rooted tree was generated with MEGA (version 7.0). Multiple sequences alignment was performed through the default parameters of ClustalW in the MEGA7 program. The phylogenetic tree was constructed using the neighbor-joining (NJ) method with the p-distance for the pairwise deletion option. The reliability of phylogenetic trees was measured by a bootstrapping method with 1,000 replicates.

### Cloning and Sequence Analysis of the Promoter

The promoter sequences (1,500 bp upstream sequences from ATG of the full-length cDNA or predicted CDS) were amplified from the PGP database. According to the plant genomic sequences, the gene-specific primers F2 (5ʹ-CCCAAGCTTAATTTATTGTCCAACAGGGG-3ʹ *Hind*III site underline) and R2 (5ʹ-CGGGATCCTCTCTTTGTAAAACTTGCCTGT-3ʹ *Bam*HI site underline) were used to amplify 1,500 bp fragment upstream of the *CaNAC035* start codon. The amplified products were re-sequenced, constructed the recombinant plasmid pBI121-CaNAC035 promoter-GUS, the result was submitted to the PlantCARE database (http://bioinformatics.psb.ugent.be/webtools/plantcare/html/) to investigate the putative *cis*-acting regulatory elements (*cis*-elements).

### RNA Extraction and qRT-PCR Analysis

The samples from six different tissues and/or organs under normal growth conditions were collected at five to six true leaves, including roots, stems, leaves, ﬂowers, fruits (about 10 days after ﬂowering), and seeds from pepper ‘P70’ to observe the expression of *CaNAC035*. For total RNA extraction, the samples were immediately frozen in liquid nitrogen. The isolation of RNA, synthesis of cDNA, and qRT-PCR were executed as described by Chen et al. (2014).

### Bioassays for GUS

For analyzing the promoter activity of *CaNAC035* under abiotic stresses, the T3 transgenic *Arabidopsis* lines were analyzed histochemically for GUS reaction. Three-week-old plant seedlings were subjected with 40°C, 40°C, 200 mM NaCl, 250 mM mannitol, 100 μM MeJA, 100 μM SA, and 50 μM ABA for 24 h, respectively. Histochemical assays for GUS reaction were performed as described in Hemerly et al. (1993) and Klahre et al. (2000) with minor modifications. The materials were washed two times with distilled water, and soaked in the solution containing 1 mM 5-bromo-4-chloro-3-indolyl-d glucuronide (X-gluc; Biosynth AG), 100 mM sodium phosphate (pH 7.0), 0.5 mM K_3_Fe(CN)_6_, 0.1% triton X-100, 0.5 mM K_4_Fe(CN)_6_, and 0.1 mM EDTA. 30 min vacuum was applied to fully infiltrate the staining solution into the plant tissues. Then, the samples were stained in GUS staining buffer at 37°C for 24 h followed by de-coloring with 75% and 95% alcohol. The samples were viewed and photographed under a microscope (Nikon, Tokyo, Japan).

### Subcellular Localization Assays

The gene-specific primers pair F3 (5ʹ-GCTCTAGAATGATCAAGGGAATCGTTGG-3ʹ *Xba*I site underline) and R3 (5ʹ-GGGGTACCAGGTTTTTGCATGTATAGGAAC-3ʹ *Kpn*I site underline) were designed by Primer Premier 5.0 software to amplify the *CaNAC035* coding region without stop codon (TGA) from the cDNA of ‘P70’ under normal growth condition. The PCR products were ﬁrst linked into a pMD-19T cloning vector (Takara), and the intermediate vector pMD-19T-*CaNAC035* was then cut by *Xba*I and *Kpn*I. The resultant digestion fragment was further inserted into pBI221-EGFP. After re-sequenced, the recombinant plasmid pBI221-*CaNAC035*-GFP was obtained.

The protoplasts of pepper were separated by enzymatic hydrolysis in the healthy ‘P70’ sterile test-tube plantlets. The 35S:*CaNAC035*-GFP and 35S:GFP (positive control) plasmids were inserted into protoplast through polyethylene glycol-mediated direct DNA transfer. The fluorescence of GFP was visualized at 488 nm by a laser confocal microscope (Nikon, Tokyo, Japan).

### Virus-Induced Gene Silencing Assay of *Canac035* in Pepper

To silence the *CaNAC035* gene, the construct pTRV2:*CaNAC035* was engineered including a 358 bp fragment of *CaNAC035* using gene-specific forward primers (5′-GCTCTAGAGAGAGTGAGAGTTTTGGGGAATT-3′ *Xba*I site underline) and reverse (5′-GGGGTACCCCTGGAAAGTGTTGAACTGGTC-3′ *Kpn*I site underline) primers. The subsequent PCR product was cloned to a vector pMD19T, the resultant construct was digested with *Xba*I and *Kpn*I, and the *CaNAC035* fragment was inserted into the *Xba*I–*Kpn*I site of pTRV2 to form the vector pTRV2:*CaNAC035*. *Agrobacterium tumefaciens* GV3101 harboring either the pTRV1 or pTRV2:*CaNAC035* were injected into pepper seedlings and the growing conditions for plants were the same as described by Wang et al. (2013).

### Generation of *Canac035* Transgenic *Arabidopsis* Plants

Gene-specific primers pair (forward, 5ʹ-GCTCTAGAGGAGATTTATTCAGACGCCTT-3ʹ; and reverse, 5ʹ-GGGGTACCGACCTACACCACCAAAGAAC-3ʹ with *Xba*I and *Kpn*I site underline) were designed to amplify the coding region of *CaNAC035*. The PCR products were digested by *Xba*I and *Kpn*I. The fragment obtained was inserted downstream of the CaMV 35S promoter of pVBG2307 to make a pVBG2307-*CaNAC035* overexpression vector. The pVBG2307-*CaNAC035* was transformed into GV3101 *Agrobacterium* strain, which was used to carry out *Arabidopsis* transformation using the floral dip method. The seeds from T0 were harvested and sown on Murashige–Skoog (MS) agar medium supplemented with 50 mg/L kanamycin and confirmed by PCR. The T3 seeds collected from independent T2 lines were used for subsequent experiments. Wild *Arabidopsis* and transgenic lines #1, #2, and #3 were selected for subsequent analysis.

### Salt and Osmotic Stress Tolerance Assays

To determine the salinity and osmotic tolerance of *CaNAC035*, seeds of WT and transgenic plants were sown on MS agar medium supplemented with NaCl (0, 150, and 250 mM) and mannitol (0, 200, and 250 mM), and assessed for seed germination (Yang et al., 2018). To determine the fresh weight, 2-week-old seedlings of WT and transgenic plants were sown on MS medium containing 150 mM NaCl or 300 mM mannitol.

### Measurement of Chlorophyll Content, Malondialdehyde, and Relative Electrolyte Leakage Assays and Antioxidant Enzyme Activities

Lipid peroxidation in the chloroplast membranes was measured in terms of malondialdehyde (MDA) produced by the thiobarbituric acid reaction (Dhindsa et al., 1981). Leaf discs from TRV2:*CaNAC035*-silenced and TRV2:00 pepper plants were collected after the cold stress-treatment, and the relative electrolyte leakage were measured as described by Dionisio-Sese and Tobita, 1998. For measuring the chlorophyll content, leaf discs from *CaNAC035*-silenced and pTRV2:00 pepper plants were collected, exposed to different concentration of NaCl and mannitol (0, 200, 300, and 400 mM each) (Zhai et al., 2017), and their chlorophyll content was measured (Arkus et al., 2005). To detect the accumulation of hydrogen peroxide (H_2_O_2_) and superoxide (O^2-^) in the transgenic and WT plants under cold stress, the 3,3ʹ-diaminobenzidine (DAB) and nitro-blue tetrazolium (NBT) staining was performed (Jabs et al., 1996; Thordal-Christensen et al., 1997). The activities of peroxidase (POD), superoxide dismutase (SOD), and catalase (CAT) were detected according to Liang et al. (2015).

### Transcriptional Activation Analysis of *Canac035*

The different truncations of *CaNAC035*, including the N-terminus domain (1–411 bp), C-terminus domain (412–921 bp), short ORF fragments (1–546, 1–600, 1–654, 1–702, 1–750, 1–777, 1–804, 1–828, 1–855, and 1–882 bp) and the full-length coding region (1–921 bp), were inserted in the pGBKT7 vectors. The positive control pGBKT7-53 + pGADT7-T and the negative control pGBKT7-Lam + pGADT7-T plasmids were transformed into Y2H yeast strain. The transformed yeast cells were coated onto SD/-Trp, SD-Ade/-His/-Trp, SD-Ade/-His/-Trp+X-α-Gal at 30°C for 3–5 days. The transcriptional activation activity of *CaNAC035* was assessed by the growth state and color of the yeast cells.

### Yeast Two-Hybrid Assays

We prepared different truncations for yeast two hybrid assays, constructs were transformed into the Y2H yeast strain. Screening for interacting proteins was performed following the manufacturer’s protocols (Clontech) with the recombinant bait plasmid with no transcriptional activity and no toxicity. Co-expression of transformed plasmids was coated onto SD/-Leu/-Trp medium. Interaction between two polypeptides was coated onto SD/-Ade/-His/-Leu/-Trp medium. Plates were incubated at 30°C until colonies became visible. The colonies which grow quickly and can turn blue were tested using T7 and 3’AD and the PCR product was used for sequencing.

### Statistical Analysis

Statistical significance was determined using a paired Student’s *t*-test (http://www.physics.csbsju.edu/stats/). The mean ± standard error (SE) from the means of three independent biological replicates and significant differences relative to controls are indicated at *P ≤ 0.05.

## Results

### Gene Sequence Analysis and Phylogenetic Tree Construction

**Figure 1:**
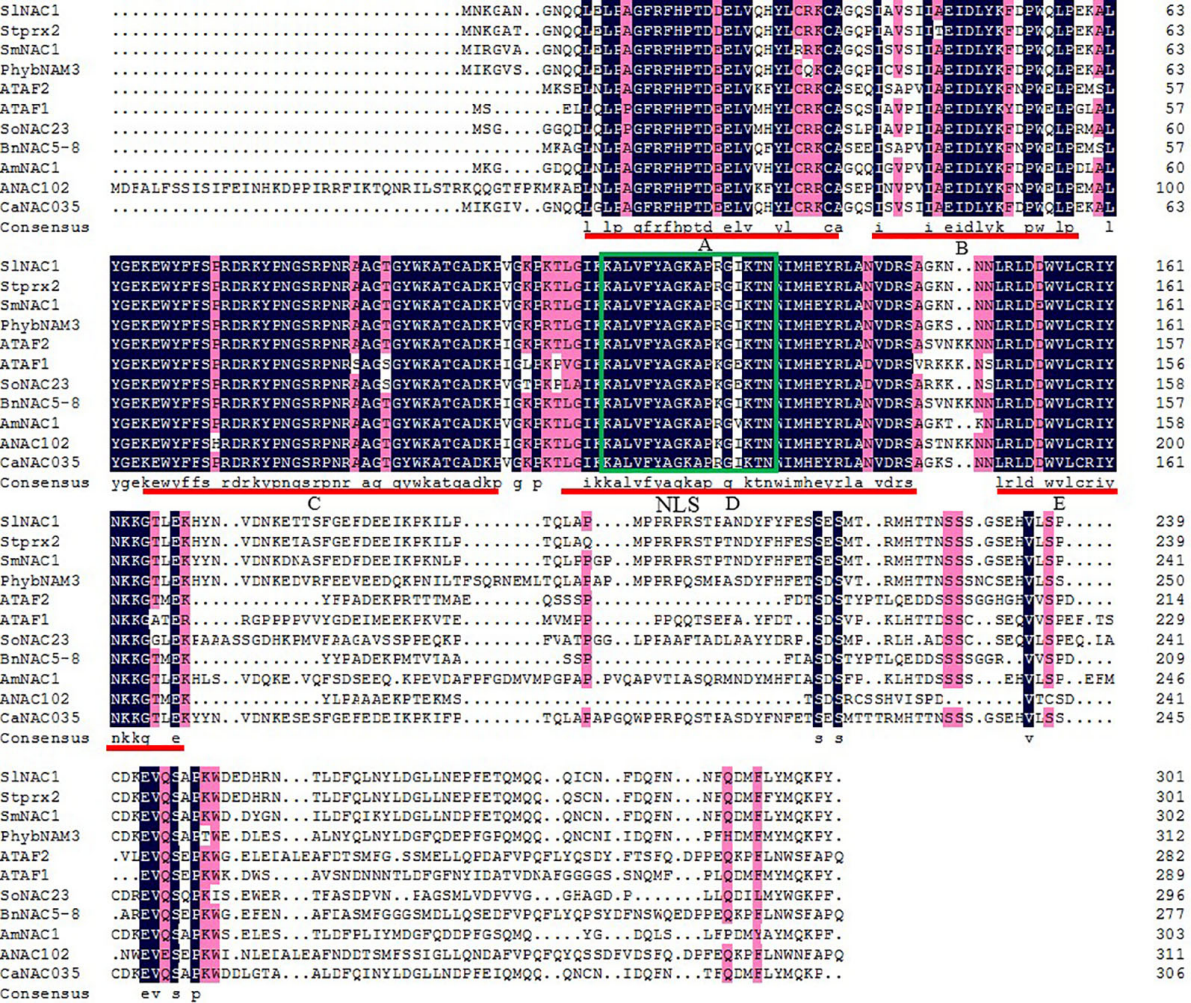
Alignment and phylogenetic analysis of CaNAC035 amino acid sequence. Multiple sequence alignment of CaNAC035 and other plant NAC members. Identical amino acids are indicated by white letters on a black background. The highly conserved region in the NAC family members is boxed. The consensus subdomains in the NAC binding domain are shown by thin underlines. The green framed part is the nuclear positioning signal (NLS).

In order to identify the structural features of *CaNAC035*, we analyzed the sequences of *CaNAC035* and predicted proteins from other species using DNAMAN software. The results showed that *CaNAC035* consists of two parts, the highly variable C-terminal region (NAM domain) and the conserved N-terminal (**Figure 1**). The NAM domain consists of five sub-domains called (A, B, C, D, and E) and the D sub-domain is a nuclear localization signal (NLS). The evolutionary relationships between CaNAC035 and other NAC proteins from different species (including *Arabidopsis*, tomato, soybean, melon, and morning glory) were evaluated *via* a phylogenetic tree which was constructed using MEGA7.0 software. The resulting tree showed that CaNAC035 is most closely related to tomato SlNAC1, and both belong to the ATAF subfamily (**Figure S1**).

### Putative *Cis*-Acting Regulatory Elements in the *Canac035* Promoter Region

To understand the mechanisms behind abiotic stress tolerance, the promoter sequence of *CaNAC035* was analyzed to find the putative *cis*-acting regulatory elements using PlantCARE database. The distributions of these elements in the promoter of *CaNAC035* are shown in the **Figure S2**. A variety of growth, development, hormone and stress responsive *cis*-acting regulatory elements were found, including hormone signaling-related elements (ABRE, ABRE3a, ABRE4, TGACG-motif, CGTCA-motif, TCA-element, and MeJA-responsive), growth- and development-related elements (GCN4_motif), light reaction-related elements (ARE, ACE, AE-box, Box 4, AT1-motif, GA-motif, I-box, G-Box, MRE), and abiotic-stress-related elements (LTR) (**Table 1**). The presence of these *cis*-elements in the promoter region suggests that the *CaNAC035* gene is actively involved in plant growth and development under stress conditions.

**Table 1:** Putative *cis*-elements identified in *CaNAC035* promoter.

Element name	Function	Element number
ABRE	*cis*-acting element involved in the abscisic acid responsiveness	6
ACE	*cis*-acting element involved in light responsiveness	1
ARE	*cis*-acting regulatory element essential for the anaerobic induction	1
AE-box	Part of a module for light response	1
AT-rich element	Binding site of AT-rich DNA binding protein (ATBP-1)	1
AT1-motif	Part of a light responsive module	1
Box 4	Part of a conserved DNA module involved in light responsiveness	1
CAAT-box	Common *cis*-acting element in promoter and enhancer regions	44
CCAAT-box	MYBHv1 binding site	1
CGTCA-motif	*cis-*acting regulatory element involved in the MeJA-responsiveness	1
G-Box	*cis*-acting regulatory element involved in light responsiveness	4
G-box	*cis*-acting regulatory element involved in light responsiveness	3
GA-motif	Part of a light responsive element	1
GCN4_motif	*cis*-regulatory element involved in endosperm expression	2
I-box	Part of a light responsive element	2
LTR	*cis*-acting element involved in low-temperature responsiveness	1
MRE	MYB binding site involved in light responsiveness	1
TATA-box	Core promoter element around -30 of transcription start	44
TCA-element	*cis*-acting element involved in salicylic acid responsiveness	1
TCT-motif	Part of a light responsive element	1
TGACG-motif	*cis*-acting regulatory element involved in the MeJA-responsiveness	1

### Analysis of *Canac035* Expression Pattern

**Figure 2:**
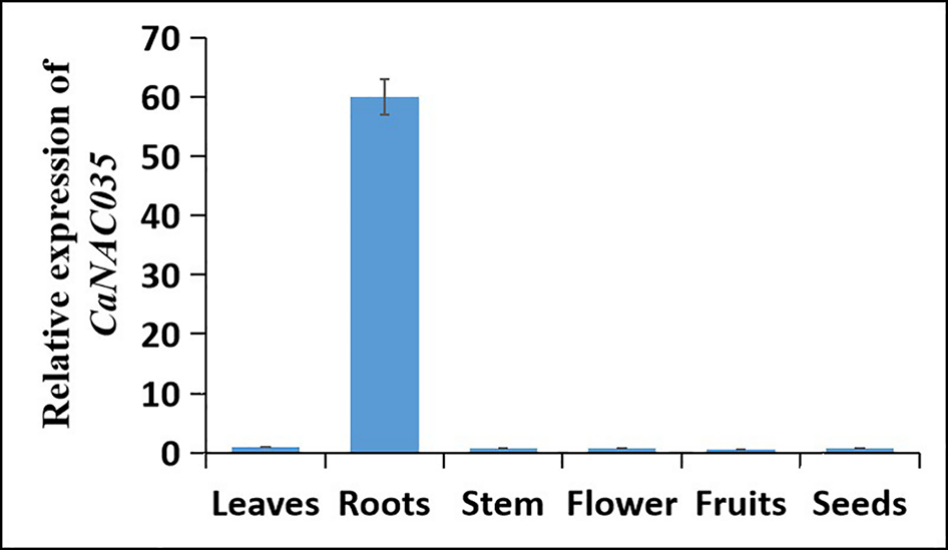
Tissue-specific expression of *CaNAC035* in seven different tissues of pepper plants. Error bars represent the mean ± SD of three independent biological replicates.

To investigate the expression of *CaNAC035* in different tissues of *C. annuum* cv. ‘P70’ plants, we performed a qRT-PCR analysis on the RNA isolated from roots, stems, leaves, flowers, fruits, and seeds. The results revealed that *CaNAC035* was constitutively expressed in all the tissues, but the expression level was the highest in the roots and the lowest in the fruits of pepper plants (**Figure 2**).

To examine the effects of abiotic stresses [cold (4°C), heat (40°C), osmotic, and salt] and phytohormone treatments (SA, MeJA, ABA, and GA) on the expression of *CaNAC035*, the ‘P70’ pepper seedlings were subjected to the abovementioned treatments, and the expression of *CaNAC035* was determined by qRT-PCR. The results depicted that *CaNAC035* was induced by cold, SA, GA, and MeJA treatments (> 20-fold). When exposed to 4°C, the *CaNAC035* transcript had no visible changes in quantity within the first 3 h and reached a maximum (~81-fold) at 24 h. For the treatments of 40°C the *CaNAC035* mRNA level peaked at 12 h after treatment (5.8 folds). After the mannitol treatment, the expression of *CaNAC035* significantly reached its highest expression level (4.8 folds) at 3 h, and then rapidly decreased after 3 h. Intriguingly, after NaCl treatment, *CaNAC035* expression rapidly reached a peak (~12.3-folds) at 48 h.

**Figure 3:**
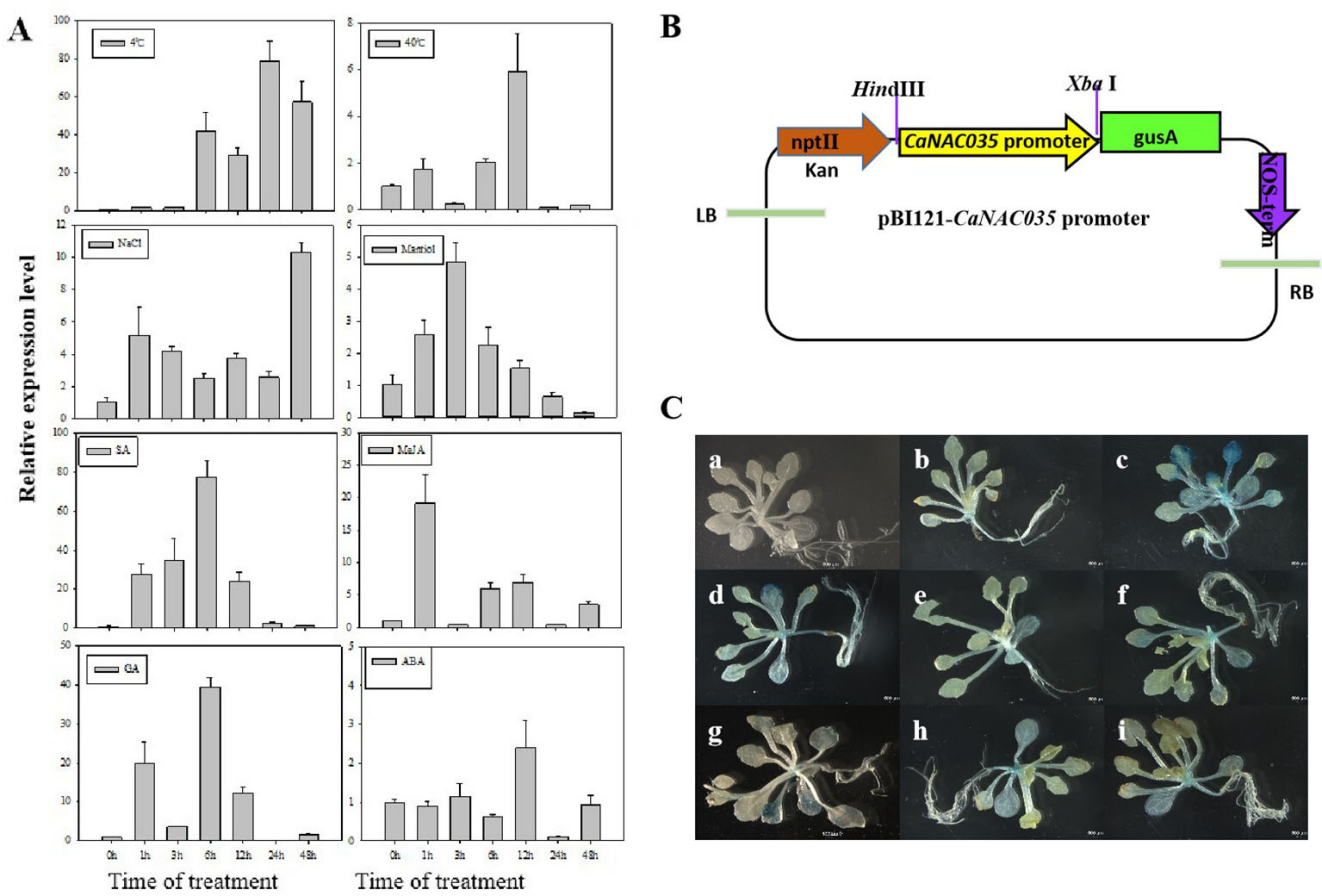
Analysis of CaNAC035 gene expression patterns. **(A)** Real-time RT-PCR analysis of *CaNAC035* expression in the leaves of pepper plants following abiotic stress and plant hormone treatments. Error bars represent the mean ± SD of three independent biological replicates. Different lower case letters indicate significant difference when compared with the control at a *p* value <0.05. **(B)** Schematic representation of the *CaNAC35* promoter::GUS construct. **(C)** Histochemical analysis of *CaNAC35* promoter::GUS transgenic *Arabidopsis*. Three-week-old plants were used to GUS staining. a, wild type; b, transgenic *Arabidopsis* under normal temperature; c, represent for low temperature for 4°C (24 h); d, 40°C (24 h); e, 200 mM NaCl (24 h); f, 250 mM mannitol (24 h); g, 100 μM MeJA (24 h); h, 100 μM SA (24 h); j, 50 μM ABA (24 h).

After GA3 treatment, the expression of *CaNAC035* reached to peak (39.8 folds) at 6 h. After ABA treatment, the *CaNAC035* transcript started to increase within 1–12 h, and its highest expression level (2.7 folds) at 12 h. After SA treatment, the *CaNAC035* transcripts had no significant changes within 3 h and then peaked (80 folds) at 12 h. Meanwhile, the expression of *CaNAC035* was also repressed by the MeJA treatment and reached its highest expression level (18.7 fold) at 1 h (**Figure 3A**).

These results suggested that the dramatic increased expression of *CaNAC035* in response to various treatments may have important implications for the stress signaling, particularly in cold stress.

To verify the expression pattern of *CaNAC035*, we carried out GUS staining analysis in the transgenic *Arabidopsis* plants expressing a *CaNAC035* promoter::GUS fusion gene construct (**Figure 3B**). **Figure 3C** shows the expression of the *CaNAC035* promoter::GUS gene in transgenic plants grown under normal growth conditions and those subjected to abiotic stresses and phytohormone treatments. Weak GUS expression was observed in several tissues of plants grown under normal conditions. In contrast, the treated transgenic plants showed strongly-induced GUS expression in several tissues, indicating that *CaNAC035* may be involved in intricate signaling pathways and can play an important role in the regulation of plant response to diverse environmental stresses.

### Subcellular Localization of Pepper Canac035

**Figure 4:**
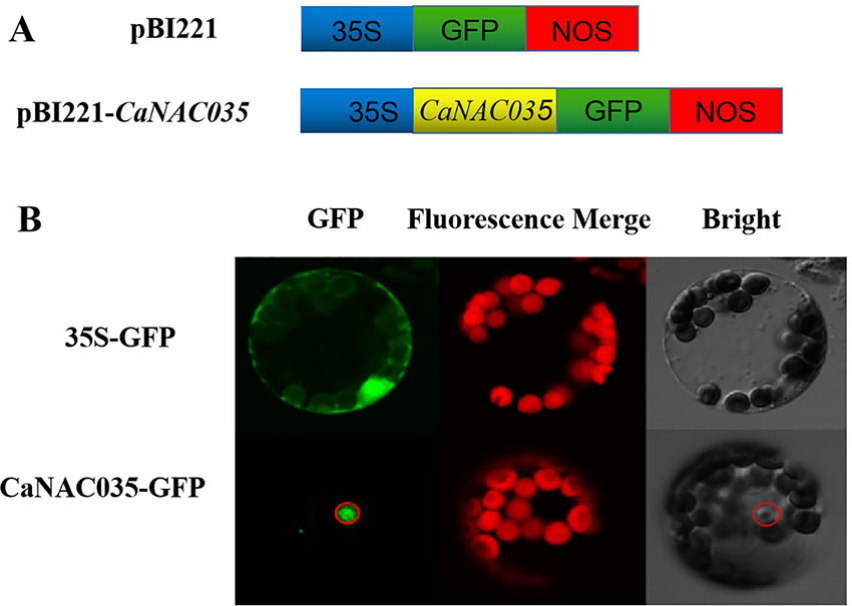
Subcellular localization of CaNAC035 protein in pepper protoplast. **(A)** Schematic representation of the CaNAC035-GFP construct. **(B)** Transient expression of GFP and CaNAC035-GFP in protoplast of pepper.

The sequence analysis of the *CaNAC035* anticipated that it contains an NLS, indicating that it may be localized in the nucleus. To test this hypothesis, the coding region of *CaNAC035* was fused in-frame to the green fluorescence protein (GFP) gene under control of the CaMV 35S promoter (**Figure 4A**). Transient expression analysis showed that the GFP signal was localized in the nucleus of pepper protoplast cells transfected with the *CaNAC035-GFP* gene fusion construct, whereas the control cells (transformed with the empty vector) had GFP ubiquitously distributed throughout the whole cell with no specific localization (**Figure 4B**). This GFP signal indicated that the CaNAC035 protein is localized in the cell nucleus.

### Suppression of *Canac035* Gene Expression Reduces Tolerance to Abiotic Stresses

To investigate the role of *CaNAC035* in the response to salt, cold, and osmotic stresses, *Tobacco rattle virus* (TRV)-based virus-induced gene silencing was performed in pepper plants ‘P70’. One month after the inoculation, the newly-emerging leaves on the top of the plant infected with pTRV2-*PDS* were completely bleached (**Figure S3A**). This silencing of phytoene desaturase showed that the virus had successfully induced gene silencing in pepper. Knockdown of the *CaNAC035* transcript levels were verified by qRT-PCR (**Figure S3B**). The results showed that the expression of the *CaNAC035* was reduced by 80–90% in new leaves of the pepper silenced plants as compared to the pTRV2:00 infiltrated control plants grown at 22°C, signifying that *CaNAC035* was successfully silenced through VIGS in pepper plants.

**Figure 5:**
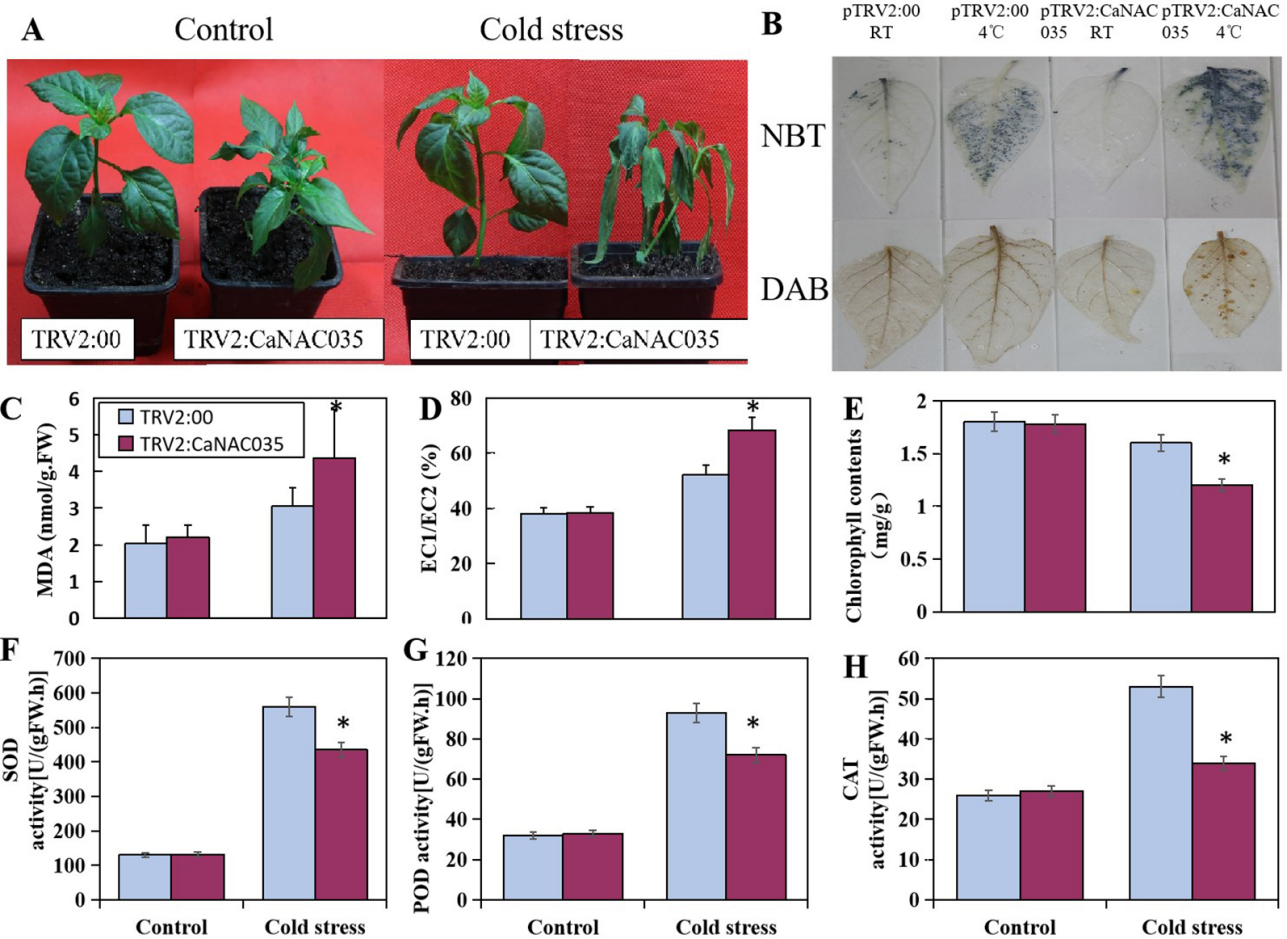
The performance of *CaNAC035*-silenced pepper seedlings under cold stress treatment. **(A)** The phenotypes of pTRV2:CaNAC035 and pTRV2-00 plants under 4°C cold stress for 48 h. **(C)** MDA levels and **(D)** electrolyte leakage in *CaNAC035-*silenced pepper seedlings. **(E)** Chlorophyll content. **(F)** SOD activity. **(G)** POD activity. **(H)** CAT activity. Asterisks indicate significant differences among the mean values ± standard error (SE) for three biological replicates based on the Student’s *t*-test, *P* ≤ 0.05. **(B)** Hydrogen peroxide and superoxide radical production in the leaves of TRV:00 and TRV-CaNAC035 plants. One month plants were treated at 4°C. The leaves were harvested after 48 h of treatment and stained with DAB and NBT.

After 48 h of 4°C cold stress treatment, the pTRV2:*CaNAC035* seedlings showed aggravated and visible symptoms of leaf damage (**Figure 5A**). There was more serious wilting in the pTRV2:*CaNAC035* pepper seedlings than in the pTRV2:00 plants after cold treatment. The electrical conductivity and MDA measurements were also significantly increased in the *CaNAC035*-silenced plants as compared to the pTRV2:00 plants (**Figures 5C, D**). The total chlorophyll content of pTRV2:00 plants was higher (~25%) than the pTRV2: *CaNAC035* silenced pepper plants (**Figure 5E**).

To determine the oxidative burst after cold stress, the concentrations of H_2_O_2_ and (O^2−^) in the leaves of *CaNAC035*-silenced and control pepper plants were measured by staining with DAB and NBT, respectively (**Figure 5B**). We observed an intense staining in the *CaNAC035*-silenced pepper plants as compared to the control plants, implying that ROS production (H_2_O_2_ and superoxide radical) were high in the *CaNAC035*-silenced plants in response to cold stress. These results indicated that *CaNAC035* is involved in the reduction of oxidative burst occurring under cold stress.

Next, we measured the activities of SOD, POD, and CAT in the pTRV2:*CaNAC035* silenced pepper plants and the pTRV2:00 plants under normal or cold conditions. In normal conditions, the activities of SOD, POD, and CAT were not significantly different between the control and silenced pepper plants. When subjected to cold stress, a remarkable increase in the activities of SOD, POD, and CAT were observed in pTRV2:*CaNAC035* and pTRV2:00 plants. However, these increases of enzymatic activities were higher in pTRV2:00 plants, as compared to the pTRV2:*CaNAC035* plants (**Figures 5F–H**).

**Figure 6:**
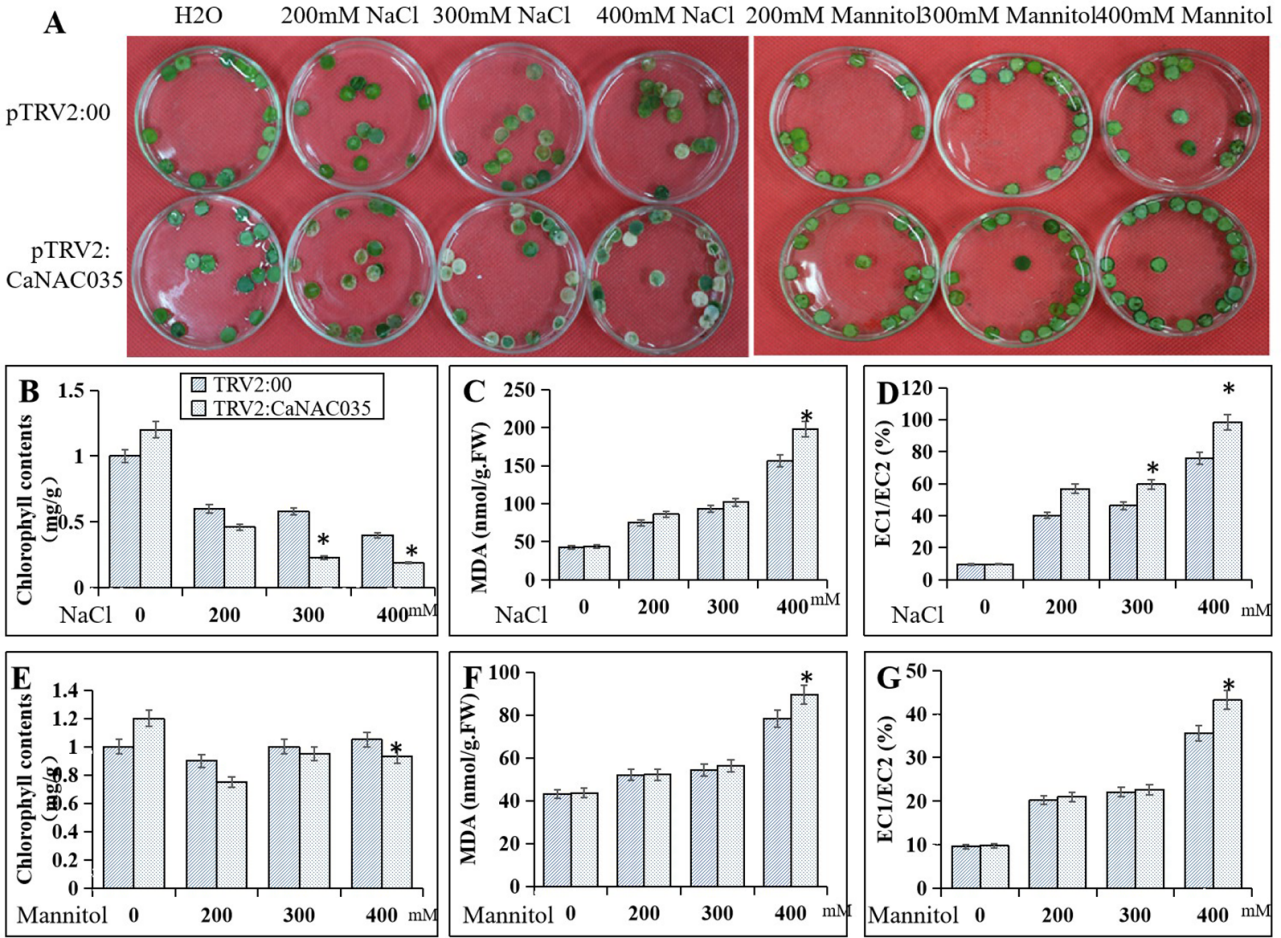
Reduced tolerance of *CaNAC035-*silenced pepper plants to salt and mannitol stress. **(A)** Phenotypes of NaCl and mannitol treatment. **(B)** Chlorophyll content after NaCl treatment. **(C)** MDA contents after NaCl treatment. **(D)** electrolyte leakage after NaCl treatment. **(E)** Chlorophyll content after mannitol treatment. **(F)** MDA content after mannitol treatment. **(G)** Electrolyte leakage after mannitol treatment. The results are the mean ± standard error (SE), replicated thrice biologically. * indicates significant differences compared with the control at a *p* value ≤ 0.05.

To ascertain the role of *CaNAC035* in the salt and osmotic stresses tolerance, leaf discs from pTRV2:00 and pTRV2:*CaNAC035* plants were exposed under continuous lighting for 3 days to 200, 300, and 400 mM NaCl and mannitol solutions, respectively. The results showed that with increasing concentrations of NaCl or mannitol, the degree of damage was more severe in the leaf discs of both the *CaNAC035*-silenced and control pepper plants. However, the *CaNAC035*-silenced leaf discs displayed more necrotic or chlorotic phenotypes than the discs of the control pepper leaves. Chlorophyll levels were also significantly reduced in the *CaNAC035*-silenced plants as compared to the control pepper plants. With the increase of NaCl and mannitol concentrations, the relative electrolyte leakage and the MDA contents were higher in the *pTRV2:CaNAC035* plants as compared to the pTRV2:00 pepper plants (**Figure 6**).

### Functional Analysis of Pepper *Canac035* in *A. thaliana*

**Figure 7:**
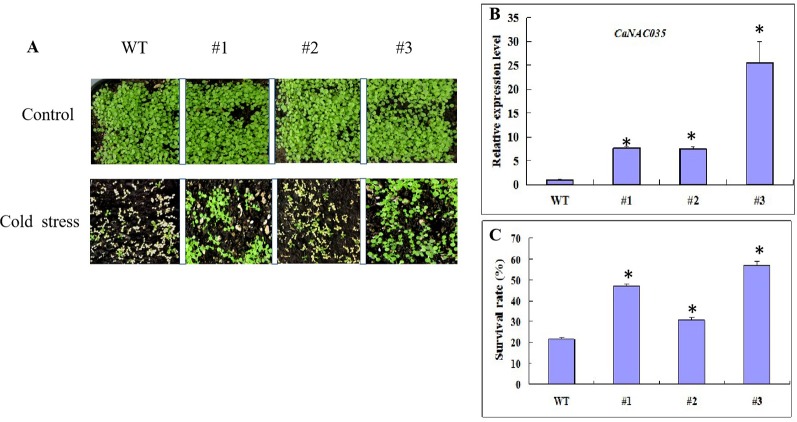
Cold tolerance analysis of WT and transgenic *Arabidopsis* plants. **(A)** Phenotypes of CaNAC035-X transgenic and WT seedlings after -20°C treatment and recovery for 7 days. **(B)** Gene expression levels of *CaNAC035* in transgenic plants and WT leaves estimated by RT-PCR (WT, #1, #2, #3). Mean values ± SE for three independent biological replicates and (*) represents the significant differences at *P* ≤ 0.05. **(C)** The percentage of surviving seedlings after cold treatment.

A construct pVBG2307-*CaNAC035* (which contained the full-length ORF of *CaNAC035* under control of the CaMV 35S promoter made for the overexpression and several overexpressed *Arabidopsis* plants (T0 generation) were generated. Five lines from the transgenic *Arabidopsis* plants were selected to examine the relative expression of *CaNAC035 via* qRT-PCR (**Figure 7B**). These results showed that the expressions of *CaNAC035* in the three lines (1, 2, and 3) were increased significantly as compared to the WT plants. Therefore, these three transgenic lines in T3-generation were selected for further experiments.

For cold stress, 3-week-old transgenic *Arabidopsis* plants and wild type from lines #1, #2, and #3 were exposed to -20°C for 20 min and then returned the plants to the standard growing conditions. After 7 days of recovery, the WT plants leaves were mostly dried and shriveled (**Figure 7A**). However, some of the transgenic plants were having green leaves and almost recovered from the cold stress. In addition, plants from the three transgenic lines had different survival rates. Transgenic line #2 had the highest survival rate of 72%, while the survival rate of the WT plants was only 19% (**Figure 7C**).

**Figure 8:**
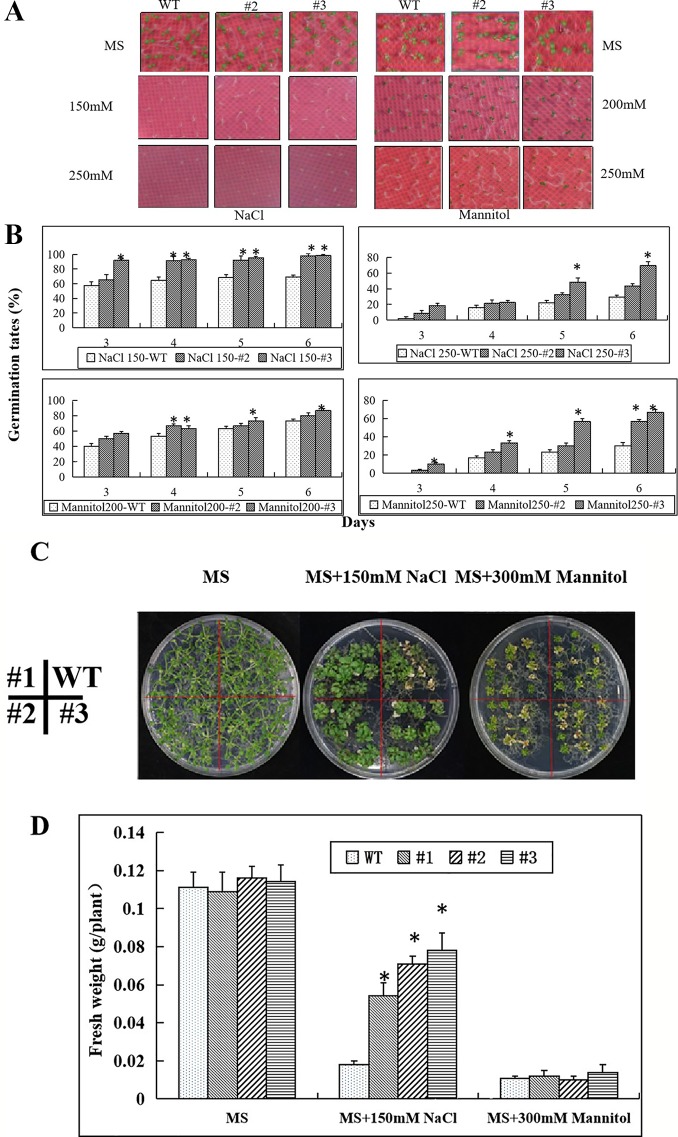
Enhanced salt and osmotic stress tolerance in seedlings of the *CaNAC035*-X transgenic *Arabidopsis* lines. **(A)** Germination of seeds of WT and transgenic plants after 7 days on MS/2 agar medium containing different concentrations of NaCl or mannitol. **(B)** Germination rates of WT and transgenic seeds exposed to different concentrations of NaCL and mannitol. **(C)** The phenotypes of wild type and transgenic *Arabidopsis* plants under salt and osmotic treatments. Four-day-old seedlings grown on 1/2MS agar medium were transferred to 1/2MS medium containing 150 mM NaCl or 300 mM mannitol. After 14 days, representative images were taken. **(D)** The seedling fresh weight were measured. Data represent the mean ± SD from three independent experiments, with 20 seeds per treatment. Asterisks above each column indicate a significant difference (*P < 0.05) between WT and the transgenic lines.

Seed germination in the transgenic lines and wild type showed no differences on MS medium without NaCl or mannitol (**Figure 8A**). However, with increasing concentrations of NaCl and mannitol, the seed germination rates gradually decreased. At concentrations of 150 mM NaCl or 200 mM mannitol, most of the seeds of the transgenic lines had germinated and some of them had yellow cotyledons, while many of the wild type seeds had not germinated. At concentrations of 250 mM NaCl and 250 mM mannitol only a few WT seeds germinated, but more than 50% of the transgenic line seeds germinated (**Figure 8B**).

Next, we transferred transgenic plants and wild type of 2-week-old seedlings onto MS medium containing 150 mM NaCl or 300 mM mannitol for salt and osmotic stress treatments. Two weeks later, we observed growth of the WT *Arabidopsis* plants was weak on the 150 mM NaCl plates (**Figure 8C**). However, there was no apparent difference between the transgenic and WT plants on the MS medium containing 300 mM mannitol. We then measured the plant fresh weights and found that the fresh weights of the transgenic plants were significantly greater than the WT *Arabidopsis* plants on the MS medium containing 150 mM NaCl. However, the fresh weights of the transgenic and WT *Arabidopsis* plants had no significant differences when grown on medium containing 300 mM mannitol (**Figure 8D**).

Taken together, these results suggested that suppressing *CaNAC035* expression reduced tolerance to cold, osmotic, and salt stresses, while overexpressing *CaNAC035* in the transgenic *Arabidopsis* enhanced the above mentioned stresses tolerance of the plants.

### Yeast Two-Hybrid Library Screening

**Figure 9:**
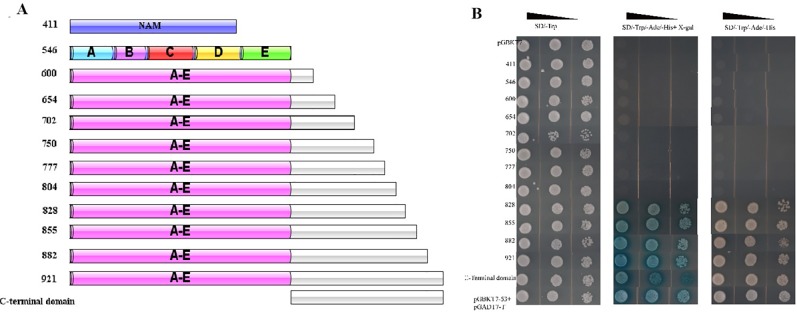
Transcriptional activation analysis of CaNAC035 and different truncations site in yeast strain Y2H. **(A)** The preliminary structure diagram of different truncated site and CaNAC035. **(B)** The result of transcriptional activation analysis. The negative control pGBKT7, the positive control P53. The culture solution of the transformed yeast was streaked on SD/-Trp medium and SD/-Ade/-His/-Trp medium. The plates were incubated for 3 days. The five conserved motifs (A to E) were analysis on the web site and sequence analysis.

To test the transcriptional activity of *CaNAC035*, we cloned several separate parts and the full-length cDNA sequence of *CaNAC035* into the pGBKT7-BD vector (**Figure 9A**). We transformed the recombinant vectors into the yeast Y2H gold cells and screened on SD/-trp, SD-Ade/-His/-Trp, and SD-Ade/-His/-Trp+X-Gal media for identification of possible self-activation (**Figure 9B**). The original figure can be found in the **Figure S4**. The results showed that recombinant vectors containing the full-length cDNA or the C-terminal region of *CaNAC035* had strong auto-activation in yeast cells. In contrast, vectors containing ORF fragments less than 804 bp in length or the N-terminal region (1–411 bp) of the *CaNAC035* gene showed no auto-activation. Therefore, the vector containing the 804 bp fragment was used as bait construct to identify proteins that potentially interact with CaNAC035. After hybridization, the blue colonies (on SD-Ade/-His/-Trp/-Leu + X-Gal medium) were picked, verified by PCR, and the inserts were sequenced using the T7 and 3ʹ-AD primers. Based on the DNA sequencing results, the 18 proteins that potentially interacted with CaNAC035 were identified by BLAST sequence searches from the NCBI database (**Table 2**).

**Table 2:** Pepper proteins identified in the yeast two-hybrid screen using CaNAC035 as bait.

No.	Gene ID	Gene annotation
1	LOC107843797	*Capsicum annuum* actin-66, mRNA
2	LOC107866727	*Capsicum annuum* transcription factor bHLH35-like, transcript variant X3, mRNA
3	LOC107855407	*Capsicum annuum* uncharacterized, mRNA
4	LOC107845643	*Capsicum annuum* protein LHCP TRANSLOCATION DEFECT, mRNA
5	LOC107862759	*Capsicum annuum* 40S ribosomal protein SA-like, mRNA
6	LOC107863177	*Capsicum annuum* uncharacterized, mRNA
7	LOC107878558	*Capsicum annuum* peptide methionine sulfoxide reductase B1, chloroplastic, mRNA
8	LOC107854570	*Capsicum annuum* 14 kDa zinc-binding protein, mRNA
9	LOC107870898	*Capsicum annuum* probable tocopherol O-methyltransferase, chloroplastic, mRNA
10	LOC107861814	Select seq ref|XP_016562669.1| PREDICTED: uncharacterized protein (*Capsicum annuum*)
11	LOC107855407	PREDICTED: uncharacterized protein (*Capsicum annuum*)
12	LOC107853693	Select seq ref|XM_016698668.1| PREDICTED: *Capsicum annuum* SNF1-related protein kinase regulatory subunit beta-2-like, mRNA
13	LOC107861808	Select seq ref|XM_016707175.1| PREDICTED: *Capsicum annuum* ruBisCO large subunit-binding protein subunit beta, chloroplastic-like, transcript variant X1, mRNA
14	LOC107866767	*Capsicum annuum* xylem cysteine proteinase 1, mRNA
15	LOC109206732	PREDICTED: *Nicotiana attenuata* putative transcription elongation factor SPT5 homolog 1, mRNA
16	LOC107864106	Select seq ref|XM_016710374.1| PREDICTED: *Capsicum annuum* 5ʹ-nucleotidase SurE-like, transcript variant X1, mRNA
17	LOC107858580	Select seq ref|XM_016703308.1| PREDICTED: *Capsicum annuum* ribulose bisphosphate carboxylase small chain 8B, chloroplastic, mRNA
18	LOC107867031	Select seq ref|XM_016713122.1| PREDICTED: *Capsicum annuum* subtilisin-like protease SBT1.2, mRNA

## Discussion

NAC transcription factors play an important role in the plant abiotic stress response. At present, more than 20 NAC family members have been identified, but studies on NAC protein functions have focused only on model plants (Morishita et al., 2009). For example, a recent study in tomato showed that the expression of *SlNAC1* was up-regulated by exogenous hormones and environmental stresses, including drought, salt, cold, ETH, SA, and ABA treatments (Ma et al., 2013). Also, suppression of *SlNAC1* reduced heat resistance in tomato plants (Liang et al., 2015). In addition, the expression of *SlNAC11* was induced significantly by cold, heat, and dehydration (Wang et al., 2018). In rice *OsNAC6* was induced by JA, ABA, wounding, and *OsNAC6*-overexpressing plants showed tolerance to cold and salt stresses (Ohnishi et al., 2005; Nakashima et al., 2007). However, overexpression of *ShNAC1* in tomato reduced drought, salt, and cold stress tolerance. In addition, the highest expression levels of *ShNAC1* were in the senescent leaves and accelerated dark- and salt-induced leaf senescence (Liu et al., 2018).

In the pepper genome there are more than 100 NAC genes and many of them show changes in expression in response to salt, cold, or drought stresses. In this study, we isolated a NAC gene from *C. annuum* leaves. Sequence analysis revealed that the predicted CaNAC035 protein consists of two parts, a highly variable C-terminal region and a conserved N-terminal region. In the conserved N-terminal region there are five sub-domains (A, B, C, D, and E) and the D subdomain functions as a NLS, which is a common feature of transcription factors. This indicated that *CaNAC035* has the conserved domains common to NAC transcription factors and subsequent subcellular localization experiments showed that it was localized in the cell nucleus. Phylogenetic analysis showed that *CaNAC035* belongs to the ATAF subfamily, which consists of TFs that play a role in tolerance to abiotic stresses such as heat, osmotic, freezing, and cold stresses (Shinozaki et al., 2003; Lata and Prasad, 2011) and it has the highest sequence homology with the tomato *SlNAC1*. It has been documented that SlNAC1 protein plays a crucial role in the abiotic stress tolerance of tomato (Huang et al., 2013). The results of this study indicated that *CaNAC035* is also involved in plant stress resistance.

In order to investigate the function of *CaNAC035* in abiotic stress response, we first determined the expression level of *CaNAC035* after exposure to abiotic stresses and exogenous hormones treatments. The results showed that lower and higher temperatures, mannitol and NaCl treatments induced the expression of *CaNAC035*. In addition, *CaNAC035* was more sensitive to ABA in phytohormone treatments and promoter activity assays (**Figure 3**). It is believed that ABA can activate the expression of transcription factors, that encode osmo-protectants including proline and hydrophilic proteins. The expression of *CaNAC035* gene was induced by exogenous SA, ABA, MeJA, and GA treatments. The results we obtained demonstrated that *CaNAC035* acted as a positive regulator to stress tolerance probably through SA, ABA, MeJA, and GA-mediated signaling pathways. These findings showed that *CaNAC035* was sensitive to exogenous SA, ABA, MeJA, and GA. We next employed VIGS to silence *CaNAC035* in the pepper and over-expression of *CaNAC035* in transgenic *Arabidopsis* plants for functional validation. In our study, when the pTRV2:*CaNAC035* gene-silenced and control plants were treated at 4°C for 48 h, the leaves damage in the gene-silenced plants was significantly more severe than in the pTRV2:00 control plants. In addition, the *CaNAC035*-silenced plants showed higher MDA content and REL. As MDA and electrolyte leakage levels reflect the degree of cellular damage the results indicated that silencing of *CaNAC035* reduced the abiotic stress tolerance of pepper seedlings. In response to cold treatment, the DAB and NBT staining showed that the gene-silenced plants had higher levels of superoxide radicals and H_2_O_2_ contents as compared to control plants. Our findings suggest that suppression of *CaNAC035* increased the oxidative stress caused by cold stress and *CaNAC035*-silenced plants suffered from more oxidative damage as compared to the control pepper plants. In addition, following NaCl and mannitol treatments, the leaf discs from *CaNAC035*-silenced plants displayed more severe injuries and low chlorophyll contents as compared to the control pepper plants. These results showed that the *CaNAC035*-silenced plants were more sensitive to osmotic, low temperature, and higher salinity stresses. In addition, the *CaNAC035* over-expressed transgenic *Arabidopsis* plants were also generated in our experiment. After 20 min of -20°C treatment followed by a 1-week recovery, we found that the survival rate of the transgenic plants were significantly higher than the WT plants. Under salt and osmotic stress conditions, seed germination rates and the fresh weights of the *CaNAC035*-overexpressed plants were significantly higher than in the WT plants. These findings indicated that *CaNAC035* confers increased tolerance to cold, osmotic, and salt stresses in pepper.

Transcription factors usually regulate the expression of multiple genes that function in concert, and the interaction between proteins is essential for the realization of protein function and to regulate biological processes (Hossain et al., 2010). To study the physical interactions between proteins *in vivo*, the yeast two-hybrid technique is an important method that can be conducted rapidly in the eukaryotic yeast cells with a high level of sensitivity (Li D.K. et al., 2014; Li X.L. et al., 2014). To further know the molecular mechanism underlying of *CaNAC035*, we performed putative protein–protein interaction with CaNAC035. A fragment of CaNAC035 was used as bait to screen for unknown protein–protein interactions. This screening generated 18 putative proteins for protein–protein interaction with CaNAC035. Bioinformatics analyses predicted these proteins participate in different processes such as the stress response, stress resistance, and photosynthesis. Our results showed that *CaNAC035* may regulate many pathways to improve plant abiotic stress resistance.

## Conclusions

In current study, the pepper *CaNAC035* gene was isolated and characterized. The predicted CaNAC035 protein has typical structural features of an NAC transcription factor and belongs to the ATAF sub-family. The CaNAC035 protein showed subcellular localization in the nucleus and it had transcriptional activity in yeast cells. Transcription of *CaNAC035* in pepper was induced by abiotic stresses and exogenous phytohormone treatments. To ascertain the role of *CaNAC035* in abiotic stress tolerance, *CaNAC035* was silenced in pepper plants through VIGS and overexpressed in *Arabidopsis*. Silencing of *CaNAC035* reduced the growth of pepper seedlings after cold, mannitol, and NaCl treatments as compared to control plants. *CaNAC035*-silenced plants showed more serious membrane damage as well as increased MDA contents after abiotic stress treatments. We also found that the *CaNAC035*-overexpressed *Arabidopsis* plants had a higher survival rate as compared to WT plants. In response to osmotic and salt stress conditions, the *CaNAC035*-overexpressed *Arabidopsis* plants exhibited higher seed germination rates and fresh weights as compared to WT plants. A yeast two-hybrid screening identified 18 proteins that potentially interacted physically *in vivo* with CaNAC035 and these potential interacting proteins participated in processes such as the stress response, stress resistance, and photosynthesis. The results of our study show that *CaNAC035* may act as a positive regulator of abiotic stress tolerance through different signaling pathways.

## Data Availability Statement

All datasets generated for this study are included in the article/**Supplementary Material**.

## Author Contributions

HZ, FM, and RC conceived and designed the experiments. HZ, FM, XH, and YZ performed the experiments. YM, US, AK, and SL analyzed the data, US reviewed and edited the manuscript. XW, DL and WZ contributed reagents/materials/analysis tools. HZ, FM, and AK wrote the paper.

## Funding

This work was supported through funding from the National Natural Science Foundation of China (#31672146, #31201615) and the Natural Science Foundation of Shaanxi Province (2018JM3023).

## Conflict of Interest

The authors declare that the research was conducted in the absence of any commercial or financial relationships that could be construed as a potential conflict of interest.
