# Supplementary material for: Molecular and Functional Characterization of CaNAC035, an NAC Transcription Factor From Pepper (Capsicum annuum L.)
Source: Front Plant Sci. 2020 Feb 4;11:14. doi: 10.3389/fpls.2020.00014 (PMC7011960; doi:10.3389/fpls.2020.00014)
Supplement: Supplementary file 1 [file DataSheet_1.docx]

Supplementary Material

Supplementary Figures 1


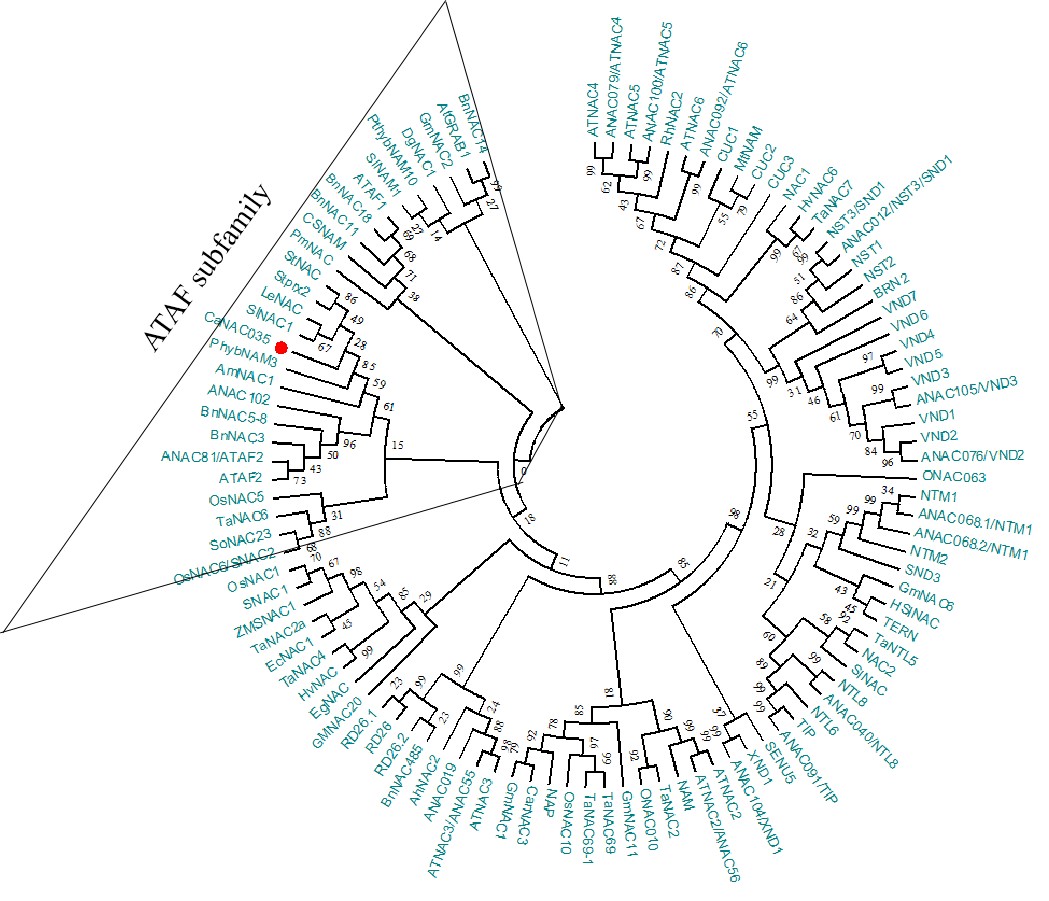


Fig. S1 Phylogenetic analysis of CaNAC035 and homologous NAC proteins from different plant species. The rooted gene tree (majority-rule consensus from 1,000 bootstrap replicates) was constructed using the heuristic searching option in MEGA7.0. Bootstrap values are indicated at each branch node. CaNAC035 was belong to ATAF subfamily and marked with red dot.

Supplementary Figures 2


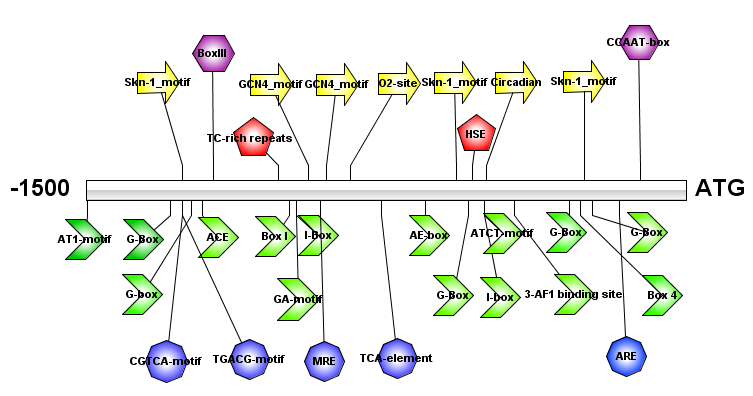
Fig. S2 Analysis of the cis-acting elements within the *CaNAC035* promoter,
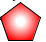
 represents the cis-acting element associated with stress,
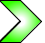
 represents cis-acting element associated with the light response,
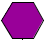
 represents site binding-related element,
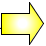
 is development-related element, and
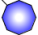
 represents hormonal response-related element.

Supplementary Figures 3


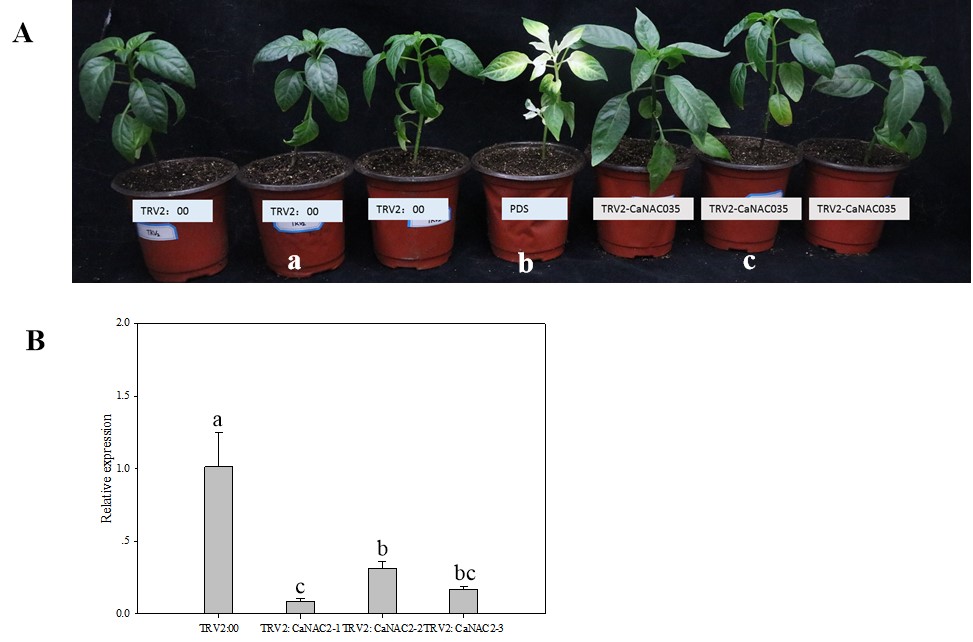
Fig. S3 VIGS of *CaNAC035* in pepper. (A) Phenotypes of gene silenced pepper plants. (a), empty-vector control (pTRV2:00); (b), *CaPDS*-silenced (TRV2:*CaPDS*); (c), *CaNAC035-*silenced (TRV2:*CaNAC035*). (B) Real-time RT-PCR analysis of *CaNAC035* expression in gene-silenced (TRV:*CaNAC035*) and control (TRV:00) plants 30 days after inoculation with the TRV vectors. The experiment was conducted with three biological replicates and each replicate contained four pepper seedlings. Error bars represent the mean ± SD of three independent biological replicates.Different lower case letters indicates significant difference when compared with the control at a *p* value <0.05.

**Supplementary Figures S4**

**
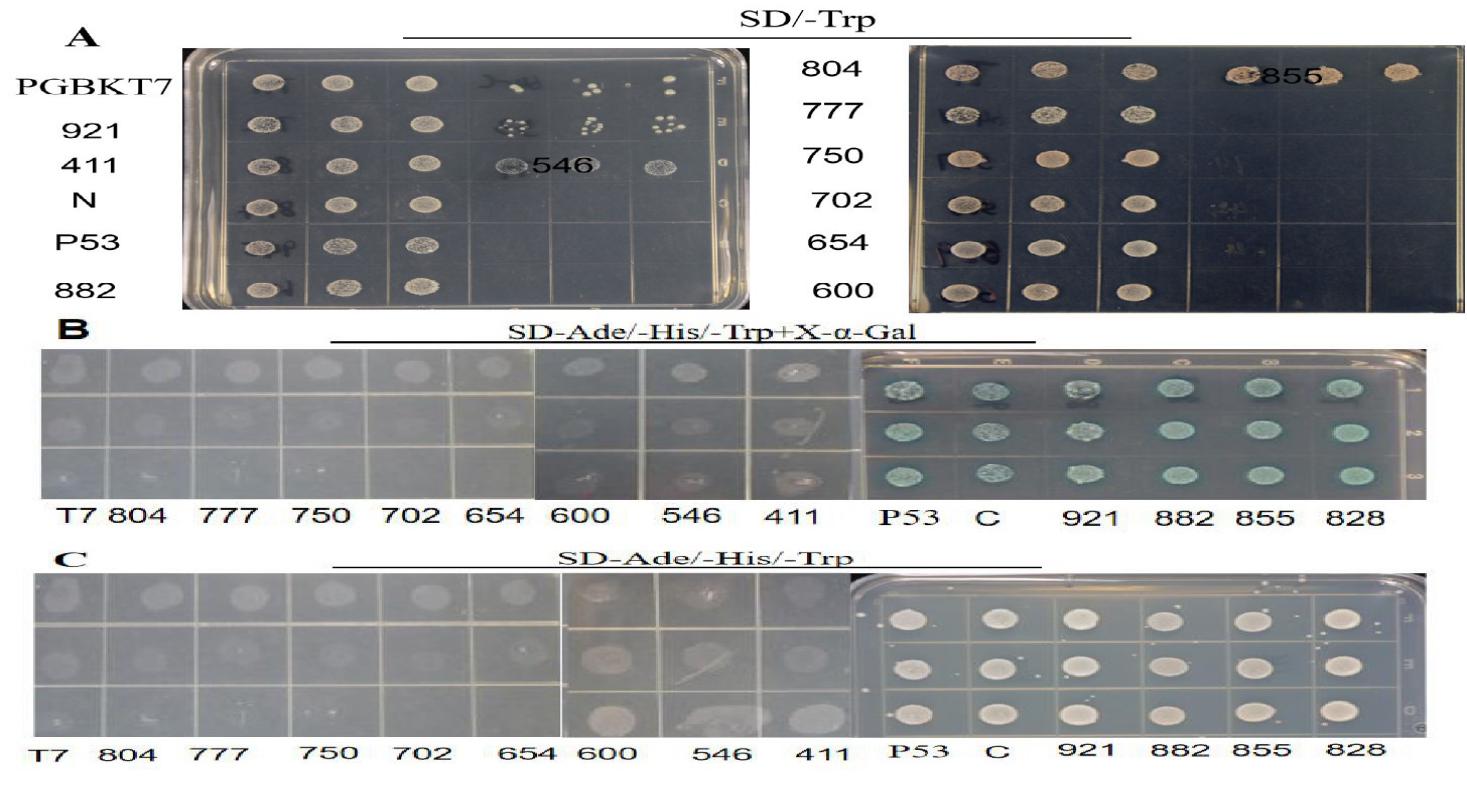
**Fig. S4 The original figure of transcriptional activation analysis of CaNAC035. A.The culture solution of the transformed yeast was streaked on SD/-Trp medium for 3 days. B. SD/-Ade/-His/-Trp+X-α-Gal medium for 3 days. C.SD/-Ade/-His/-Trp medium for 3 days.
